# Inhibition of *MEIS3* Generates Cetuximab Resistance through c-Met and Akt

**DOI:** 10.1155/2020/2046248

**Published:** 2020-12-08

**Authors:** Ping Cai, Yangyang Xie, Mingjun Dong, Qiaoqiao Zhu

**Affiliations:** ^1^Anorectal Surgery, Hwa Mei Hospital, University of Chinese Academy of Sciences, 41 Northwest Street Road, Haishu District, Ningbo 315800, China; ^2^Ningbo Institute of Life and Health Industry, University of Chinese Academy of Sciences, 41 Northwest Street Road, Haishu District, Ningbo 315800, China; ^3^Key Laboratory of Diagnosis and Treatment of Digestive System Tumors of Zhejiang Province, 41 Northwest Street Road, Haishu District, Ningbo 315800, China; ^4^Department of Medical Experiment, Hwa Mei Hospital, University of Chinese Academy of Sciences, 41 Northwest Street Road, Haishu District, Ningbo 315800, China

## Abstract

**Introduction:**

Although cetuximab has been widely used in the treatment of colon cancer, a large number of patients eventually develop drug resistance. Therefore, it is essential to clarify the mechanism of drug resistance.

**Methods:**

In this study, we combined in silico analysis and a single guide RNA (sgRNA) library to locate cetuximab-sensitive genes. Cell proliferation, apoptosis, and cell cycle were assessed to validate the change in cetuximab sensitivity. Finally, western blotting was performed to detect changes in epidermal growth factor (EGFR) upstream and downstream genes.

**Results:**

Using in silico analysis and the sgRNA library, *MEIS3* was confirmed as the cetuximab-sensitive gene. Further experiments indicated that the expression of *MEIS3* could determine the level of cetuximab. Meanwhile, *MEIS3*-inhibited cells were sensitive to mesenchymal epithelial transition factor (c-Met) and protein kinase B (Akt) inhibitors, which is related to the change in phosphorylation of c-Met and degradation of Akt.

**Conclusion:**

*MEIS3* modified the sensitivity to cetuximab through c-Met and Akt.

## 1. Introduction

In China, the number of newly diagnosed colorectal cancer cases reached 432,000, making colorectal cancer one of the most commonly diagnosed cancers [1]. In stage I patients, the 5-year survival rate is approximately 90%; however, when metastasis occurs, the survival rate drops to 14% [2]. To prolong the survival rate of the patients, multiple studies have attempted to clarify the critical pathway in the proliferation and metastasis of colon cancer. Currently, the target pathways that have been discovered include vascular endothelial growth factor (VEGF) [3], VEGFR [4], EGFR [5], and B-Raf (BRAF) [6].

Cetuximab is an inhibitor of EGFR, which is used to treat metastatic colon cancer, non-small-cell lung cancer, and head and neck cancer [7]. In July 2009, cetuximab was approved by the Food and Drug Administration (FDA) for the treatment of colon cancer with wild-type Kirsten ras sarcoma viral oncogene (*KRAS*) [8]. Thirty-one clinical trials have thus far been conducted for the treatment of metastatic colon cancer patients including cetuximab as monotherapy or in combination with chemotherapy [9]. Concurrently, the drug resistance to cetuximab has also increased; the mechanism of cetuximab resistance involves mutation in the *KRAS* gene [10], activation of upstream genes such as *C-MET* [11], and upregulation of the downstream genes such as *AKT* [12]. In this study, we attempted to use a high-throughput screening approach to locate genes that are responsible for cetuximab resistance. The MEIS3 gene is a relatively new gene, which encodes a homeobox domain, so it can bind to DNA and regulate the binding of specific DNA sequences, promoter transcription activity, and so on [13]. In mice, MEIS3 gene expression can regulate the expression of protein 3-phosphatidylinositol-dependent protein kinase-1 [14]. In the mesoderm stage, the change of the MEIS3 expression level can significantly affect the activity of the Wnt signaling pathway and determine the fate of cells. However, the relationship between the MEIS3 gene and cetuximab resistance has not been reported [15].

## 2. Materials and Methods

### 2.1. In Silico Analysis

The in silico analysis in this study was completed using the R 3.5.1 software. The cetuximab-sensitive genes were identified in the following manner. (1) We first extracted the drug screen data from the Sanger Institute (https://www.cancerrxgene.org/), and among these datasets, 43 colon cancer cell lines were selected and ranked by the half-maximal inhibitory concentration (IC50) of cetuximab. (2) The top three and the lowest three cell lines were selected, and the RNA-seq data of these six cell lines were extracted. (3) The RNA-seq reads were first normalized by log_2_ transformation, and the differentially expressed genes between IC50 high and low cell lines were calculated using the limma package (linear models for microarray data); 933 genes with FC (fold change) < −2 and *P* value < 0.05 were identified as downregulated genes. (4) The sgRNA synthesis and library readout were completed by the BGI Group (Beijing Genomics Institute, China). (5) Regarding The Cancer Genome Atlas (TCGA) database, the RNA-seq and clinical data from TCGA-Colon Adenocarcinoma (TCGA-COAD) dataset were first downloaded using the GDCRNATools package, and then, the relationship between the expression of *MEIS3* and patients' overall survival rate was detected using the survival package.

### 2.2. Patient Samples

Seventeen cetuximab-sensitive and nine cetuximab-resistant tumor samples were obtained from the Ningbo Beilun People's Hospital. All the patients included in this study signed an informed consent, and the ethics committee of the Ningbo Beilun People's Hospital approved all the procedures, and the approval number is YJ-KYSB-NBEY-2018-028-01.

### 2.3. Cell Culture and Transfection

Colon cancer cell lines CaR-1, CCK-81, SUN-61, CL-40, LOVO, HT-29, KM-12, CL-11, LS-180, LS-513, and MDST8 were purchased from ATCC company, and all the cell lines above were cultured in Dulbecco's modified Eagle medium (DMEM)+10% fetal bovine serum (FBS) medium. The cultures were maintained at 37°C with 5% CO_2_. shRNA and cDNA used in this experiment were synthesized by BGI (Beijing Genomics Institute) company. The transfection of vector was performed using Lipofectamine 2000 reagent (Thermo Fisher Scientific, US); in the transfection, Lipofectamine reagent and vector were first diluted by Opti-MEM medium and then mixed together and cultured for 10 min in room temperature; DNA-lipid complex was then added to cells, and the transfection procedure was finished. And for the cell line experiment, LOVO cells were divided into the LOVO-CON and LOVO MEIS3 shRNA groups, then transfected with CON and MEIS3-shRNA vectors for 48 h, and served for subsequent experiment. In the meantime, SNU-61 cell lines were divided into the CON and MEIS3 groups and transfected with CON and MEIS3 overexpression vectors for 48 h.

### 2.4. Quantitative Reverse Transcription PCR (qRT-PCR)

#### 2.4.1. mRNA Extraction

First, the tumor tissues from cetuximab-resistant and cetuximab-sensitive patients were homogenized and dissolved in TRIzol reagent. The solution was then mixed with chloroform to separate the mRNA, which was then precipitated using isopropanol and washed with 75% ethanol.

#### 2.4.2. Reverse Transcription

mRNA was then reverse transcribed using the PrimeScript RT Reagent Kit with gDNA Eraser (RR047B, Takara Company, Dalian, China). The transcription process was performed according to the instructions provided by the manufacturer.

#### 2.4.3. qPCR

After cDNA was obtained using glyceraldehyde-3-phosphate dehydrogenase (GAPDH) as the control, the relative expression of *MEIS3* was quantified using the TB Green Advantage qPCR premixes (639376, Takara Company, Dalian, China). The quantification method used was 2^−*ΔΔ*Ct^, and the primer sequence was GAPDH F: 5′-GTCTCCTCTGACTTCAACAGCG-3′ and R: 5′-ACCACCCTGTTGCTGTAGCCAA-3′ and *MEIS3* F: 5′-ATCATGCGAGCCTGGTTGTTCC-3′ and R: 5′-CATAGGTTGCACGATGCGTCTC-3′.

### 2.5. Western Blot

The western blot was performed in the following manner. (1) The cells were first dissolved in radioimmunoprecipitation assay (RIPA) buffer on ice for 30 min; the loading buffer was then added and boiled for 15 min. (2) The sample was loaded onto the sodium dodecyl sulfate-polyacrylamide gel electrophoresis (SDS-PAGE) to separate the protein by molecular weight. (3) The proteins were then transferred to polyvinylidene fluoride (PVDF) membranes and blocked with 5% bovine serum albumin (BSA) for two hours. (4) The membranes were labeled with the first antibody, which included the following: GAPDH (Cat No. 5174), Akt (Cat No. 4691), p-Akt (Cat No. 4060), c-Met (Cat No. 8198), phospho-c-Met (Cat No. 3077), EGFR (Cat No. 2085), and p-EGFR (Cat No. 2244), which were all purchased from Cell Signaling Technology (MA, USA). MEIS3 (Cat No. PA5-61288) antibody was purchased from Thermo Fisher Scientific (Waltham, MA, USA). (5) The membranes were labeled with horseradish peroxidase (HRP) secondary antibodies, which were all purchased from Zhongshan Jinqiao Biotechnology Co., Ltd. (Beijing, China), and finally, the protein expression was detected using SuperSignal HRP Chemiluminescent Substrates (Thermo Fisher Scientific, MA, USA).

### 2.6. Drug Treatment

For this study, cetuximab (A2000), crizotinib (S1068), and miltefosine (S3056) were all purchased from Selleck Chemicals Llc (Houston, Texas, USA). The concentration of drugs that were used for treatment is described in Results. The drug library was designed and completed by Prof. Xingguo Zhang.

### 2.7. Cell Proliferation, Apoptosis, and Cell Cycle

#### 2.7.1. Cell Proliferation

The cells were first plated on a 24-well plate and were treated with the MTT reagent subsequent to cell attachment. The plate was then washed twice with phosphate-buffered saline (PBS), 0.1% MTT was added, and the cells were cultured at 37°C for 20 min. The plate was then washed with PBS, and isopropanol was added. Finally, cell proliferation was determined at optical density (OD) 450 nm.

#### 2.7.2. Apoptosis

After the cetuximab treatment, the cells were harvested and washed twice with PBS, then suspended in binding buffer, and labeled with Annexin V APC antibody (Sanjian Company, Tianjin, China). Prior to detection using the fluorescence-activated cell sorting (FACS) technique, the cells were labeled with propidium iodide (PI) to identify the dead cells.

#### 2.7.3. Cell Cycle

After cetuximab treatment, the cells were harvested, washed with PBS, and were fixed in 70% ethanol overnight. The fixed cells were treated with RNase and labeled with a high concentration of PI for 20 min. Finally, the cell cycle was detected by FACS.

### 2.8. Statistical Analysis

The statistical analysis was performed using the R 3.5.1 software with Student's *t*-test, and the criterion of statistical significance was *P* value lower than 0.05. And repetitive data in this paper was shown as median ± SD.

## 3. Results

### 3.1. Identification of *MEIS3* as a Cetuximab-Sensitive Gene

**Figure 1:**
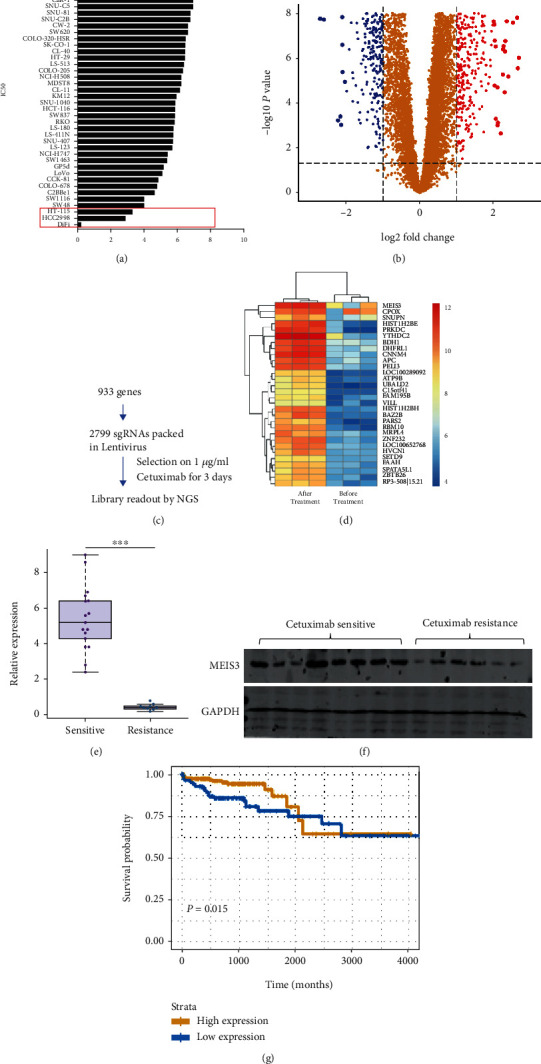
(a) Based on the IC50 of cetuximab in 43 colon cancer cell lines from the Genomics of Drug Sensitivity in Cancer (GDSC) dataset, we selected SNU-C1, CL-34, and SW1417 as cetuximab-resistant cell lines and HT-155, HCC2998, and DiFi-09844799147 as cetuximab-sensitive cell lines. (b) The RNA expression profile in both cetuximab-sensitive and cetuximab-resistant cells was obtained, and genes with low expression in cetuximab-resistant cell lines were identified. (c) Pipeline of transfection of sgRNA library and drug selection. (d) NGS readout was used to quantify sgRNA. (e) mRNA from bulk tumor tissue was extracted from 17 cetuximab-sensitive and nine cetuximab-resistant patients, and expression of *MEIS3* was identified by qPCR. (f) Bulk tumor tissue protein from eight cetuximab-sensitive and six cetuximab-resistant cell lines was extracted, and the expression of MEIS3 was detected by western blot (*n* = 3). (g) The relationship between the expression of MEIS3 and the overall survival rate of COAD patients in TCGA database.

In this study, we first extracted drug-sensitive information from the Sanger Institute. Forty-three colon cancer cell lines with different IC50 values were identified in the cetuximab treatment dataset. After their ranking by the IC50 data, we chose the top three cell lines as cetuximab-resistant cell lines and the last three cell lines as cetuximab-sensitive cell lines (Figure 1(a)). Using limma to analyze the transcriptional data, we identified 933 genes that were downregulated in cetuximab-resistant cell lines (Figure 1(b)). Then, the 933 genes were assigned to 2799 sgRNAs and transfected into CaR-1, CL40, and KM-12 cells. Cells were selected with cetuximab, and next-generation sequencing (NGS) was used to readout the library representation. We confirmed that sgRNA targeting *MEIS3* had the highest enrichment ratio among the 2799 sgRNAs (Figures 1(c) and 1(d)). Using our in-house data, we detected the expression of *MEIS3* in 17 cetuximab-sensitive and nine cetuximab-resistant patients and confirmed that *MEIS3* had a low expression in all the cetuximab-resistant samples at the mRNA level (Figure 1(e)). Further, *MEIS3* had a low expression in six cetuximab-resistant samples at the protein level (Figure 1(f)). In TCGA-COAD database, the expression of *MEIS3* at the mRNA level was highly correlated with the overall survival rate of the colon cancer patients (Figure 1(g)). Considering these results, we confirmed that *MEIS3* is a cetuximab-sensitive gene.

### 3.2. Manipulation of Cetuximab Sensitivity by *MEIS3*

**Figure 2:**
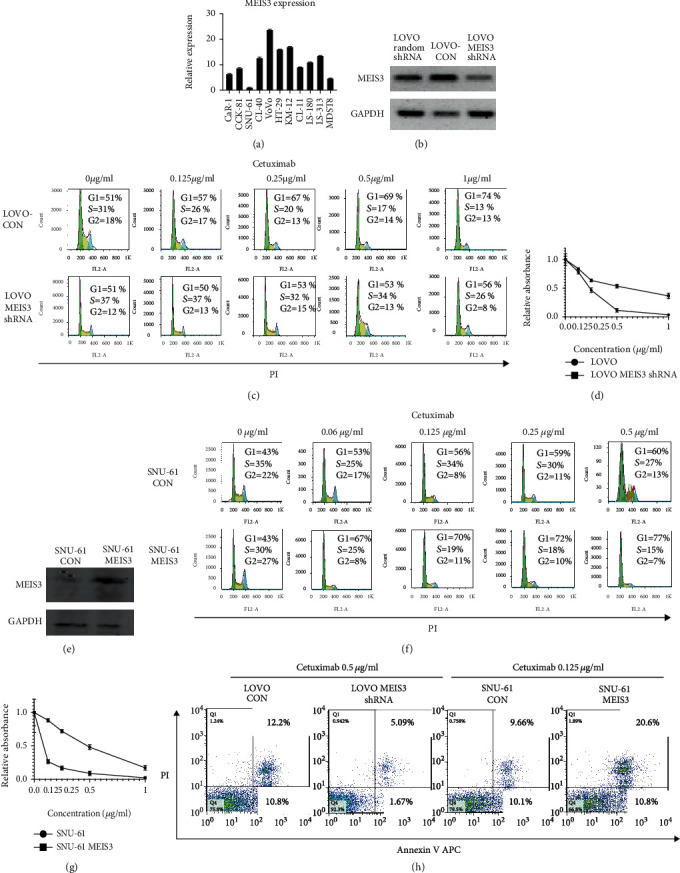
(a) mRNA was extracted from 11 colon cancer cell lines, and the expression of MEIS3 was detected by western blot (*n* = 3). (b) The LOVO cells were transfected with random shRNA, CON vector, and *MEIS3* shRNA, and the knockdown efficiency was confirmed by western blot after 48 h. (c) The LOVO-CON and *MEIS3* shRNA cells were treated with different concentrations of cetuximab for 48 h, and PI staining was used to detect changes in the cell cycle. (d) The LOVO-CON and *MEIS3* shRNA cells were treated with different concentrations of cetuximab for 48 h, and the cell viability was detected using an MTT assay. (e) The SNU-61 cells were transfected with CON and *MEIS3* cDNA, and the expression of MEIS3 at the protein level was detected by western blot after 48 h (*n* = 3). (f) The SNU-61 CON and MEIS3 groups were treated with different concentrations of cetuximab for 48 h, and PI staining was used to detect changes in the cell cycle. (g) The SNU-61 CON and MEIS3 groups were treated with different concentrations of cetuximab for 48 h, and cell viability was detected using an MTT assay. (h) The LOVO and LOVO *MEIS3* shRNA cells were treated with 0.5 *μ*g/ml cetuximab for 48 h while the SNU-61 and SNU-61 MEIS3 were treated with 0.125 *μ*g/ml cetuximab for 48 h, and the change in apoptosis was detected using the Annexin V/PI double staining.

To further clarify the role of *MEIS3* in cetuximab sensitivity, we analyzed 11 colon cancer cell lines in our lab; QPCR confirmed that SNU-61 had the lowest expression of *MEIS3*, while LOVO had the highest expression (Figure 2(a)). We hence designed *MEIS3* shRNA and transfected into LOVO cells considering that *MEIS3* shRNA could knockdown MEIS3 protein level (Figure 2(b)); LOVO-CON and LOVO *MEIS3* short hairpin RNA (shRNA) were treated with different doses of cetuximab for 48 h; as the concentration of cetuximab was increased in the CON group, the G1 ratio of LOVO cells increased, while in the *MEIS3* shRNA cells, the increase in the G1 ratio was slower than that in the CON group, which indicated that in *MEIS3*-inhibited cells, the G1 arrest induced by cetuximab is alleviated (Figure 2(c)). In the MTT assay, after treatment with 0.5 *μ*g/ml and 1 *μ*g/ml cetuximab, the survival rate of the *MEIS3* shRNA group was significantly higher than that of the CON group (Figure 2(d)). Using SNU-61, which is another *MEIS3* low-expression cell line, we further confirmed that overexpression of *MEIS3* not only promoted G1 arrest induced by cetuximab (Figures 2(e) and 2(f)) but also inhibited the growth of SNU-61 cells (Figure 2(g)). Furthermore, after treatment with cetuximab, apoptosis of LOVO *MEIS3* shRNA was significantly lower than that of LOVO, and SNU-61 *MEIS3* was significantly higher than that of SNU-61 CON (Figure 2(h)). Thus, the expression of *MEIS3* could determine cetuximab sensitivity.

### 3.3. Inhibition of p-EGFR Was Delayed When *MEIS3* Was Downregulated

**Figure 3:**
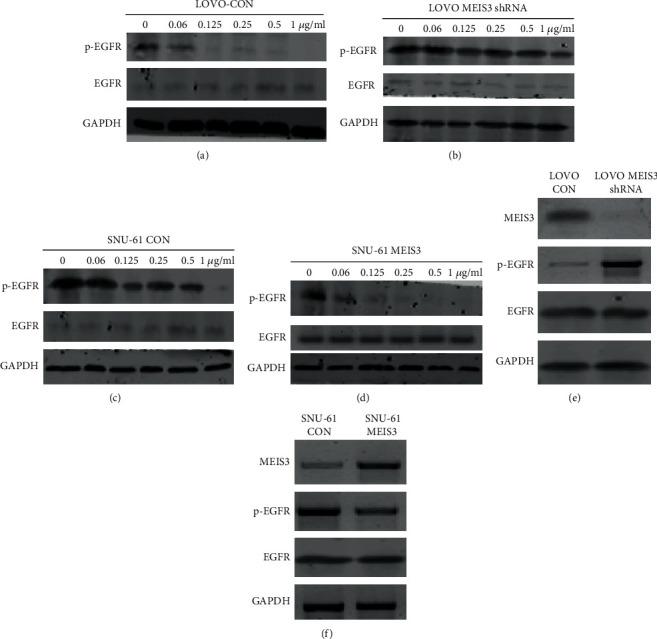
Colon cancer cell lines (a) LOVO-CON, (b) LOVO *MEIS3* shRNA, (c) SNU-61 CON, and (d) SNU-61 MEIS3 were treated with different concentrations of cetuximab for 48 h. The expression of EGFR and p-EGFR was detected by western blotting (*n* = 3). (e) In LOVO-CON and *MEIS3* shRNA cells, expression of MEIS3, p-EGFR, and EGFR was detected by western blot (*n* = 3). (f) In SNU-61 CON and SNU-61 MEIS3 cells, the expression of MEIS3, p-EGFR, and EGFR was detected by western blot (*n* = 3).

Since the mechanism of action of cetuximab is the inhibition of EGFR phosphorylation, we investigated the changes in EGFR phosphorylation. As previously described, when LOVO-CON and *MEIS3* shRNA were treated with different concentrations of cetuximab, EGFR phosphorylation in the LOVO-CON group decreased dramatically after treatment with 0.125 *μ*g/ml cetuximab; however, EGFR phosphorylation in the *MEIS3* shRNA group did not change until treatment with 1 *μ*g/ml cetuximab, which is further indicated by gray intensity ratio (gray intensity ratio in p-EGFR/gray intensity ratio in GAPDH); in LOVO-CON, gray intensity ratio in p-EGFR was 0.29, 0.10, 0.05, 0.03, 0.02, and 0.001, and in LOVO-MEIS3 shRNA, it is 1.52, 1.63, 1.32, 1,18, 1.13, and 0.75 (Figures 3(a) and 3(b)). When *MEIS3* cDNA was transfected into the SNU-61 cell line, the phosphorylation of EGFR in the CON group was inhibited after treatment with 1 *μ*g/ml cetuximab, while in the *MEIS3* group the phosphorylation of EGFR was inhibited after treatment with 0.25 *μ*g/ml cetuximab (Figures 3(c) and 3(d)). These results indicate that the expression of *MEIS3* correlated with the phosphorylation of EGFR. When *MEIS3* was inhibited in LOVO cells, the phosphorylation of EGFR increased while the total EGFR was unchanged (Figure 3(e)). Likewise, when *MEIS3* was overexpressed, phosphorylation of EGFR was decreased (Figure 3(f)). Based on these results, we concluded that *MEIS3* could control the phosphorylation of EGFR.

### 3.4. Using Drug Library to Overcome Cetuximab Resistance

**Figure 4:**
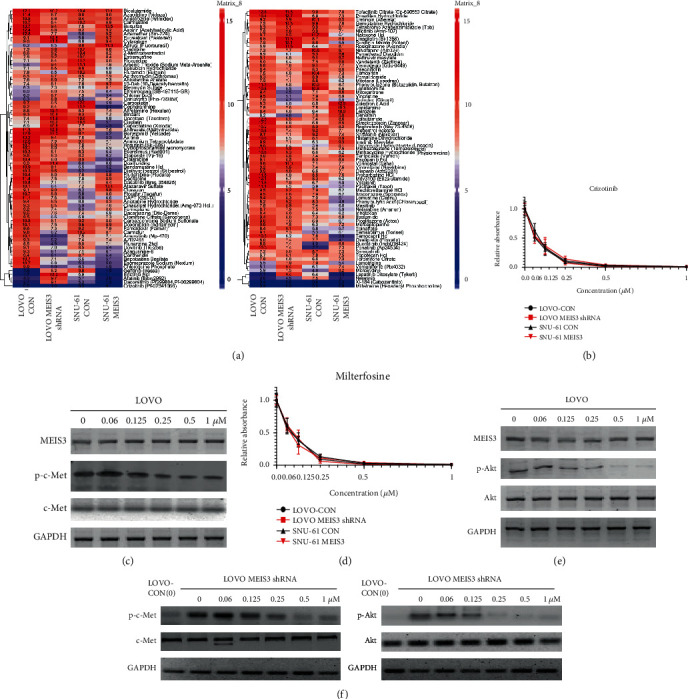
(a) The IC50 pattern of LOVO-CON, LOVO *MEIS3* shRNA, SNU-61 CON, and SNU-61 MEIS3. (b) The LOVO-CON, LOVO *MEIS3* shRNA, SNU-61 CON, and SNU-61 MEIS3 cells were treated with different concentrations of crizotinib, and the proliferation of cells was detected using an MTT assay. (c) The LOVO cells were treated with different concentrations of crizotinib, and the expression of MEIS3, p-c-Met, and c-Met was determined by western blot (*n* = 3). (d) The LOVO-CON, LOVO *MEIS3* shRNA, SNU-61 CON, and SNU-61 MEIS3 cells were treated with different concentrations of miltefosine, and cell proliferation was determined using an MTT assay. (e) The LOVO cells were treated with different concentrations of miltefosine, and the expression of MEIS3, p-Akt, and Akt at the protein level was determined by western blot (*n* = 3). (f) LOVO-CON and LOVO *MEIS3* shRNA cells were treated with different concentrations of crizotinib and miltefosine, and expression of MEIS3, p-Akt, and Akt at the protein level was determined by western blot (*n* = 3).

Considering that the inhibition of *MEIS3* could introduce cetuximab resistance, we used high-throughput approaches to overcome this resistance; a drug library containing 146 drugs was applied, and each drug was assigned at five concentrations. By calculating IC50, we found that LOVO-CON, LOVO *MEIS3* shRNA, SNU-61 CON, and SNU-61 *MEIS3* were all sensitive to inhibitors of c-Met such as crizotinib and XI-184 as well as to miltefosine, which is an inhibitor of Akt (Figure 4(a)). To validate our theory, we again treated the cells in 24-well plates, which further confirmed that LOVO-CON, LOVO *MEIS3* shRNA, SNU-61 CON, and SNU-61 *MEIS3* were all sensitive to both crizotinib (Figure 4(b)) and miltefosine (Figure 4(d)). Consistent with this result, the phosphorylation of c-Met was decreased after crizotinib treatment (Figure 4(c)), and phosphorylation of Akt additionally decreased after treatment with miltefosine (Figure 4(e)). Furthermore, after MEIS3 is inhibited in LOVO cell lines, p-c-Met and p-Akt are upregulated as expected; however, the elevated p-c-Met and p-Akt have still been inhibited by crizotinib and miltefosine treatment, suggesting that crizotinib and miltefosine served as powerful inhibitors of c-Met and Akt (Figure 4(f)). Concurrently, *MEIS3* was unchanged during both drug treatments; considering that the c-Met pathway could regulate the Akt pathway, we suspected that *MEIS3* might locate on the upstream of Akt and c-Met.

### 3.5. Regulation of Phosphorylation of c-Met and Degradation of Akt by *MEIS3*

**Figure 5:**
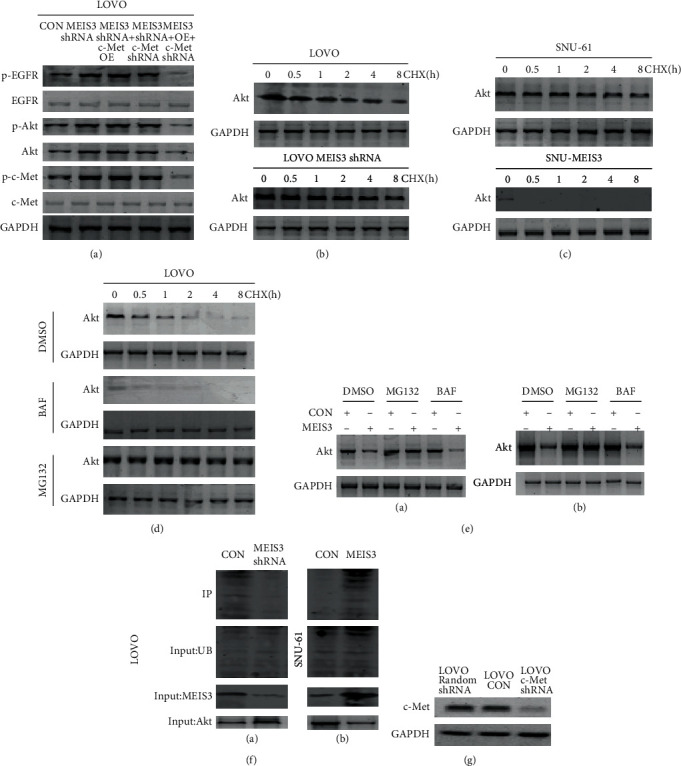
(a) Protein levels of LOVO-CON, *MEIS3* shRNA, *MEIS3* shRNA+c-Met overexpression, *MEIS3* shRNA+c-Met shRNA, and MEIS3 overexpression+c-Met shRNA were extracted, and the expression of p-EGFR, EGFR, p-Akt, Akt, p-c-Met, and c-Met at the protein level was detected by western blot. (b) The LOVO and LOVO *MEIS3* shRNA were treated with CHX for different times, and the expression of Akt was detected by western blot. (c) The SNU-61 cell line was treated with CHX for different times, and the expression of Akt was detected by western blot. (d) The LOVO cells were first treated with CHX followed by DMSO, BAF, and MG132 for different times, and the expression of Akt was determined by western blot. (e) SNU-61 (A) and HCT-116 (B) were transfected with CON and *MEIS3* shRNA and then treated with DMSO, MG132, and BAF, and the degradation of Akt was determined by western blot. (f) LOVO (A) and SNU-61 (B) were transfected with the CON and MEIS3 vectors, and the ubiquitin-bonded Akt was detected using IP. (g) The LOVO cell line was transfected with random shRNA, CON vector, and c-Met shRNA for 48 h, and then, western blot was used to detect the change of c-Met in the protein level.

Both crizotinib and miltefosine could overcome the cetuximab resistance caused by *MEIS3* inhibition; however, these two drugs could not change the expression of *MEIS3*. This indicated that these 2 drugs might be concentrated on MEIS3 downstream pathways; given that crizotinib and miltefosine are targeting c-Met and Akt, we suspected that *MEIS3* might be able to regulate the c-Met and Akt pathways. Therefore, we constructed LOVO-CON, *MEIS3* shRNA, *MEIS3* shRNA+c-Met OE (overexpression), *MEIS3* shRNA+c-Met shRNA, and *MEIS3* OE+c-Met shRNA cell lines. Western blotting indicated that when c-Met was overexpressed, the phosphorylation of EGFR reached the highest level among these five groups, and when c-Met was inhibited, the phosphorylation of EGFR decreased. Concerning Akt, the inhibition of *MEIS3* increased the phosphorylation of Akt and total Akt compared with the *MEIS3* shRNA+c-Met OE group thereby suggesting that the expression of Akt could be regulated by *MEIS3.* Concurrently, the phosphorylation of c-Met was increased after *MEIS3* was inhibited, which indicated the MEIS3-c-Met-EGFR axis (Figure 5(a)). As the expression of total Akt is increased after *MEIS3* is inhibited, we first treated LOVO-CON and LOVO *MEIS3* shRNA with cycloheximide (CHX) and then harvested the protein at different time points. By detecting the change in Akt, we concluded that the degradation of Akt was inhibited after *MEIS3* expression was decreased (Figure 5(b)). In the *MEIS3* OE cells, degradation of Akt was highly promoted (Figure 5(c)). When treated with the autophagy inhibitor bafilomycin A1 (BAF), the expression of Akt decreased even after CHX treatment. Using the ubiquitination inhibitor MG132, the degradation of Akt was prevented, which indicated that the degradation of Akt was completed by the ubiquitination pathway (Figure 5(d)). Similarly, in SNU-61 and HCT-116 cell lines, transfection of *MEIS3* cDNA increased the degradation of Akt, which was stopped by MG132 (Figure 5(e)). Furthermore, using immunoprecipitation (IP) experiments we confirmed that when *MEIS3* is inhibited, ubiquitin-bonded Akt is decreased (Figure 5(f)). These results show that *MEIS3* could regulate the phosphorylation of c-Met; further, ubiquitination of *AKT* is also regulated by *MEIS3.*

## 4. Discussion

Although the application of cetuximab has been confirmed, a phenomenon of cetuximab resistance has been identified. The mechanism of resistance to cetuximab is closely related to its mechanism of action. The known mechanisms include (1) binding to the EGFR receptor on the surface of tumor cells with high affinity and competitively blocking the binding of EGF and EGFR, thus inhibiting the expression of downstream genes; (2) binding to the Fc fragment on the surface of immune cells to induce cell-mediated cytotoxicity; and (3) inducing EGFR into the cell and reducing the phosphorylation level of EGFR, thus inhibiting the proliferation and metastasis of tumor cells [10]. However, most patients will develop drug resistance after 6–12 months of cetuximab treatment; it is thus essential to understand the underlying mechanism to overcome this resistance [16]. The mechanism of cetuximab resistance includes primary and secondary resistance. In primary resistance, patients develop drug resistance during the first treatment. It accounts for 60% of the total incidence of resistance to the drug [17]. Most of the primary resistance was due to the abnormal activation of EGFR downstream or upstream genes such as *KRAS* [18], *PI3K/AKT* [19], and *IGF1* [20]. Part of the cetuximab resistance was due to compensation by the NF-*κ*B pathway [21]. Most of the secondary resistance was due to mutations in *EGFR* [22].

In this study, we first identified genes that were downregulated in cell lines that are less sensitive to cetuximab using sgRNA screening. We confirmed that after cetuximab treatment, sgRNA targeting *MEIS3* was highly expressed in surviving cells; further proliferation, apoptosis, and cell cycle analysis concluded that the expression of *MEIS3* could change cetuximab sensitivity. MEIS3 belongs to the MEIS family of proteins, which forms dimeric or trimeric DNA-binding complexes with PBX and/or HOX proteins [23] and is widely involved in cell functions such as neural stem cell development [24] and hematopoiesis [23]. Unlike MEIS1 and MEIS2, the function of MEIS3 is not well known. To clarify and overcome cetuximab resistance caused by *MEIS3* inhibition, we applied a drug library containing 146 drugs. Through drug screening, we identified that the *MEIS3* shRNA cell line was sensitive to c-Met and Akt pathway inhibitors. Further analysis indicated that *MEIS3* could alter the phosphorylation of EGFR and Akt, and most importantly, *MEIS3* could change the ubiquitination of Akt.

## 5. Conclusion

Our results indicate that *MEIS3* functions as a cetuximab-sensitive gene, which works through the phosphorylation of c-Met and degradation of Akt.

## Data Availability

The data used to support the findings of this study are available from the corresponding author upon request.
